# Nurses’ perspective about the Mental Health First Aid Training Programmes for adolescents in upper secondary schools: A focus group study

**DOI:** 10.1111/jpm.12823

**Published:** 2022-02-08

**Authors:** Tiago Filipe Oliveira Costa, Francisco Miguel Correia Sampaio, Carlos Alberto da Cruz Sequeira, María Teresa Lluch Canut, Antonio Rafael Moreno Poyato

**Affiliations:** ^1^ Centro Hospitalar de Vila Nova de Gaia / Espinho Vila Nova de Gaia Portugal; ^2^ Nursing School of Porto Porto Portugal; ^3^ 16724 Department of Public Health Mental Health and Maternal and Child Health Nursing Nursing School Universitat de Barcelona Hospitalet de Llobregat Spain; ^4^ “NursID: Innovation & Development in Nursing” of the Center for Health Technology and Services Research (CINTESIS) Porto Portugal; ^5^ Higher School of Health Fernando Pessoa Porto Portugal; ^6^ GEIMAC (Consolidated Group 2014‐1139: Group of Studies of Invarianza of the Instruments of Measurement and Analysis of Change in the Social and Health Areas) Barcelona Spain

**Keywords:** adolescent, education, first aid, mental health, nursing, qualitative research

## Abstract

**What is known on the subject?:**

- Mental Health First Aid Training Programmes with a pathogenic perspective are implemented worldwide for different participants and contexts. These interventions can promote the medicalization and psychiatrization movement of human suffering.
- Training programmes should teach about mental health nursing problems rather than disorders. However, there seem to be no studies describing these healthier interventions targeting adolescents in upper secondary schools. Nurses can explore these interventions and target them towards these participants and contexts.

**What the paper adds to existing knowledge?:**

- The perspective of nurses on the characteristics of Mental Health First Aid Training Programmes for adolescents in Portuguese upper secondary schools is reported.
- Experts recognize that the nurses who perform these interventions must have personal, pedagogical and mental health competencies. Therefore, mental health nurses may be considered. The components of mental health literacy, mental health nursing problems and a dynamic first aid plan can be taught using different classroom training strategies.

**What are the implications for practice?:**

- The role of mental health nurses in promoting health literacy is highlighted. They have the opportunity to lead multidisciplinary teams in using these healthier training programmes.
- These expert opinions can shape the planning, implementation and evaluation of these interventions. In turn, training programmes can promote the identification, assistance and/or adequate and timely referral of people with mental health nursing problems.

**Abstract:**

**Introduction**

Mental Health First Aid Training Programmes performed by nurses can empower adolescents to aid people with mental health problems. There do not appear to be any studies that describe these healthier interventions aimed at adolescents in upper secondary schools. The development of these educational interventions benefits from the input of their participants and facilitators.

**Aim**

To explore the perspective of nurses about Mental Health First Aid Training Programmes for adolescents in upper secondary schools.

**Method**

A qualitative, descriptive and exploratory study was conducted. Data were collected from seven mental health nurses during two focus group sessions. Data were analysed using content analysis.

**Results**

Twelve themes were identified around nine main areas: facilitators, intervention foci, outcomes assessment methods, process assessment methods, participants, implementation context, duration and frequency, intervention methods and strategies, and contents.

**Discussion**

Mental health nurses can regularly perform these classroom interventions to improve the mental health competencies of adolescents. Various educational methods can facilitate learning related to mental health literacy components, mental health nursing problems and the first aid plan. Valid and appropriate assessment methods enhance a good representation of these interventions.

**Implications for Practice**

This evidence can guide the creation and modelling of these first aid training programmes.

## 1 INTRODUCTION

Mental health problem is a broad term that includes both mental disorders and the symptoms of a mental disorder that do not yet justify the diagnosis of a disorder (Kitchener et al., 2017). The high prevalence of mental health problems increases the likelihood of people having contact with individuals with these problems (Morgan et al., 2018). The general public could provide first aid until they receive professional help or the crisis is resolved (Kitchener et al., 2017).

First aid provision requires people to take an active role in the health of others. In turn, citizens can become more actively involved in their personal and community health, improving their health literacy (World Health Organization [WHO], 2016). Mental health literacy comprises five components: knowledge of how to prevent mental disorders, recognition of when a disorder is developing, knowledge of help‐seeking options and treatments available, knowledge of effective self‐help strategies, and knowledge and skills to provide first aid and support others (Jorm, 2012). The levels of mental health literacy are low worldwide (Tay et al., 2018). For example, pioneering studies by Loureiro et al. (2013, 2015) supported this trend in Portuguese youth. Therefore, Mental Health First Aid Training Programmes are important. These educational interventions aim to disseminate basic first aid skills in the community (Kitchener & Jorm, 2017). These programmes involve the training of individuals to aid people with mental health problems. They do not include the therapist's direct actions towards people with mental health problems and should not be confused with Crisis Interventions.

Nurses are professionals who have been reported as vital in promoting health literacy (World Health Organization [WHO], 2018). Costa et al. (2020) suggest that nurses develop these training programmes, addressing mental health nursing problems. These authors expressed three premises to support this recommendation. Firstly, existing first aid training programmes seem to respond positively to nursing foci (relevant care areas for nurses). Second, specialized training and experience in mental health, psychoeducational skills and proximity to the implementation context are characteristics of the facilitators of these interventions that can be found in mental health nurses. Finally, existing Mental Health First Aid Training Programmes in the literature mainly address pathologies. Therefore, these interventions can promote the medicalization and psychiatrization movement of human suffering because their participants view mental health problems as disorders (DeFehr, 2016). Therefore, training programmes with a healthier perspective should be considered. For instance, the nursing diagnosis of "anxiety" can be addressed instead of the medical diagnosis of "anxiety disorder."

These interventions that address psychiatric terms and structures have been carried out for adolescents in the upper secondary schools. For example, Hart et al. (2016), Hart et al. (2020) and Guajardo et al. (2019) describe positive results of classroom‐based Mental Health First Aid Training Programmes in students aged 15 to 18. In this way, these participants and contexts are suitable targets of this type of intervention. The school is identified as an appropriate context to intervene in the literacy promotion (Directorate‐General for Health [DGS], 2018; McDaid, 2016). It encompasses people from a wide range of ages and with diverse economic, social and cultural characteristics. In upper secondary education, students are expected to acquire multiple factual and theoretical knowledge, cognitive and practical skills necessary to perform tasks and solve problems, assume responsibilities and adapt behaviour to circumstances (European Commission, 2018). In Portugal, upper secondary school students (10th, 11th and 12th grades) are normally aged 15 to 18 (European Commission, 2021). People aged 10 to 19 are called adolescents (Organisation for Economic Co‐operation and Development [OECD] / World Health Organization [WHO], 2020). Adolescents are expected to have logical and systematic reasoning and hypothetical‐deductive thinking, which enable the assimilation and accommodation of learning (Halpenny & Pettersen, 2014). During adolescence, people also reflect on the roles they are and will be playing in the world, achieving a sense of identity (Ferrer‐Wreder & Kroger, 2020). Thus, this seems to be an important phase for them to incorporate the role of “first aider.”

In an exploratory search, studies describing these healthier interventions for adolescents in upper secondary schools were not found. Therefore, the opportunity to develop this type of training programme for these specific participants and contexts was identified. This development process can be guided by the structure of the UK Medical Research Council (Richards & Hallberg, 2015). One of the steps includes modelling the interventions, and their potential participants and facilitators should be considered (Richards & Hallberg, 2015). Costa et al. (2021) explored adolescents’ perspectives on Mental Health First Aid Training Programmes promoted by nurses in Portuguese upper secondary schools. Adolescents confirmed the relevance of these interventions and provided guidelines for their content and intervention strategies (Costa et al., 2021). However, it is still necessary to explore the perspective of the potential facilitators (nurses).

## 2 AIM AND RESEARCH QUESTION

This study aims to explore the perspective of nurses about Mental Health First Aid Training Programmes for adolescents in Portuguese upper secondary schools. The research question is “What is the opinion of nurses about the characteristics of Mental Health First Aid Training Programmes for adolescents in upper secondary schools?”. The characteristics of intervention include their facilitators, foci, outcomes and process assessment methods, participants and specific implementation context, duration and frequency, intervention methods and strategies, and contents.

## 3 METHODS

### 3.1 Design

We carried out a qualitative, descriptive and exploratory study. The study report was guided by the *Consolidated criteria for reporting qualitative research (COREQ) checklist* (Tong et al., 2007).

### 3.2 Selection of participants

Participants were mental health nurses with work developed in mental health literacy with adolescents aged 15 to 18 and in the Portuguese school context. Mental health nurses have been described by Costa et al. (2020) as nurses who should preferably perform these interventions. In addition, expertise in the research topic may be reflected in more thoughtful and appropriate opinions about the topic.

Participants were selected using a non‐probabilistic, intentional sampling method. In this way, people with particular characteristics and the potential to provide rich, relevant and diverse data on the subject under study were involved (Robinson, 2014). The researchers discussed the potential participants and their expertise, mainly considering their productivity and the quality of their work (Caley et al., 2013). No relationship with the participants had been established before the beginning of the study.

The size of the expert panel usually varies from four to twelve, with the ideal number of participants being five to eight (Krueger & Casey, 2015). Due to the lack of guarantee of the attendance of potential participants, Nyumba et al. (2018) recommend over‐recruiting 10–25%. Therefore, ten potential experts were invited, eight confirmed their presence, and seven finally participated. The potential participants who declined the invitation and those who withdrew from the study indicated personal reasons to justify their decisions.

Potential participants were invited, via e‐mail, to participate in the study voluntarily and without any compensation. They were asked to fill out (online) an informed consent, a sociodemographic characterization form and a survey of availability to meet. Then, focus group sessions were scheduled.

### 3.3 Data collection

Focus groups were held. This technique is based on the notion that group interaction encourages respondents to explore and clarify individual and shared perspectives (Morgan, 1997). Data were collected in the native language of the researchers and study participants (European Portuguese).

The interview guide was developed in response to the objective of the study and the research questions (Kallio et al., 2016). The interview guide was pretested by applying it to three mental health nurses. The nurses reported that the interview guide was understandable, clear, unambiguous, well‐structured, without bias and did not need any changes. Supporting information S1 includes the interview guide used.

In July 2021, the first author conducted two focus group sessions, and the second author supervised them. These took place by videoconference (using the Zoom platform), allowing easier, more flexible and safer access to experts from different parts of Portugal. Each meeting lasted approximately two hours. Audio and video recording of the experts´ meetings were carried out to promote reliable reporting of the content discussed. The recordings were transcribed immediately after the meetings. The transcripts were returned to the participants for comments and/or corrections.

The characteristics of this type of intervention were discussed until participants did not produce new information and a clear pattern of responses was reached. Therefore, data saturation was obtained (Saunders et al., 2018).

### 3.4 Data analysis

The collected data were subjected to content analysis (Graneheim & Lundman, 2004; Graneheim et al., 2017). Thus, the transcriptions of the recordings were considered units of analysis. The text under analysis was classified deductively in the following content areas: facilitators, intervention foci, outcomes and process assessment methods, participants and specific implementation context, duration and frequency, intervention methods and strategies, and contents of training programmes. The content analysis was performed by the first author and verified by the other authors. There were no disagreements in the process, and no software was used.

Firstly, the text under analysis was read several times to get a sense of the whole. Then, the text was divided into meaning units. In turn, the meaning units were condensed and labelled with a code. The various codes were compared based on their differences/similarities and classified into subcategories and categories. Finally, the underlying meanings of the categories formed subthemes and themes at an interpretive level. The participants provided positive feedback on the results.

### 3.5 Ethical considerations

In the present study, all ethical assumptions in the Declaration of Helsinki (World Medical Association, 1964) and the Oviedo Convention (Resolution of the Assembly of the Republic No (2001) No. 1/2001) for research with human beings were fulfilled. The ethics committee of the University of Barcelona approved the research proposal to carry out the study (Institutional Review Board: IRB00003099).

Informed consent was obtained from all participants. They were notified of the right to voluntarily participate and withdraw from the study at any time without penalty. Moreover, they were advised of the right to access, rectify, limit processing and delete their data.

Each expert's data were coded with the letter E and a number to preserve confidentiality. Furthermore, the data were stored in a computer and external disc (with login credentials) used exclusively for this study.

## 4 RESULTS

Seven experts participated in the focus group sessions. Most mental health nurses were female and had children (86%). They were aged 36 to 56 years old (mean: 45.29, SD: 6.97). In addition, 86% of participants were married or in non‐marital partnership, and 14% were divorced or separated. Participants had different academic degrees (29% Licentiate, 29% Master and 43% Doctorate). Most participants performed more than one role in nursing (86%). They played roles in care provision (71%), management (14%), education (86%) and research (71%). Participants were nurses in different regions of Portugal (43% in the North, 43% in the Centre and 14% in the South). Their experience as non‐specialized nurses ranged between 5 and 14 years (mean: 10.14, SD: 3.72) and as mental health nurses between 6 and 23 years (mean: 13.29, SD: 6.40).

Twelve themes were identified in response to the research questions (Table 1). The codes and categories that support each theme can be found in Supporting information S2.

**TABLE 1:** Themes obtained from content analysis

Content Areas	Themes obtained
Intervention Facilitators	Theme 1: Mental health nurses with co‐dynamization of other professionals
Intervention Foci	Theme 2: Mental health competence
Outcomes assessment methods	Theme 3: Longitudinal evaluation with validated instruments based on knowledge in the nursing field
Process assessment methods	Theme 4: Valid and adapted assessment of satisfaction for each training session
Participants	Theme 5: Implementation for classes with a nursing diagnosis, availability and willingness to learn
Specific implementation context	Theme 6: Classroom implementation during school time
Duration and frequency	Theme 7: Regular sessions depending on school availability
Intervention methods and strategies	Theme 8: Mixture of different training strategies and educational resources Theme 9: Awareness sessions for the school community
Contents	Theme 10: Components of mental health literacy Theme 11: Mental health nursing problems Theme 12: Dynamic mental health first aid plan

### 4.1 Intervention facilitators

**FIGURE 1:**
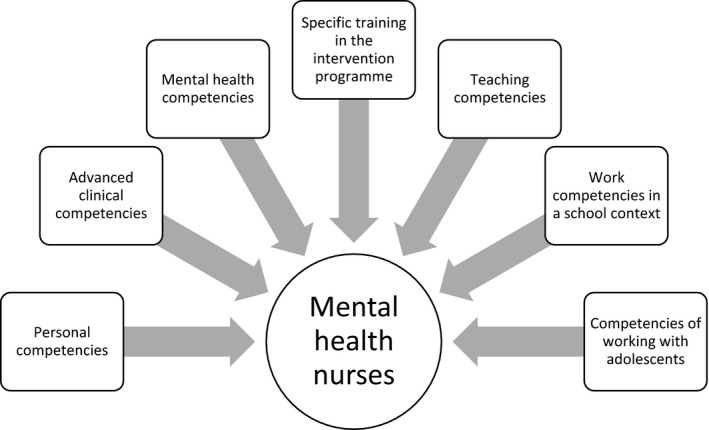
Characteristics of nurses who perform these interventions

According to the experts, these training programmes are likely to be carried out by “Mental health nurses with co‐dynamization of other professionals.” They reported different characteristics that nurses who implement the intervention must have (Figure 1). The profile described can be found in mental health nurses.

Although a facilitator can perform the intervention, the experts valued the co‐dynamization of the intervention. E2 declared that “It is important to have co‐facilitators because they support the implementation and evaluation of interventions.” Therefore, they recommended the inclusion of professionals with different training (health professionals from different disciplines and specialities, education professionals) and from distinct contexts (primary and hospital care).

### 4.2 Intervention foci

**FIGURE 2:**
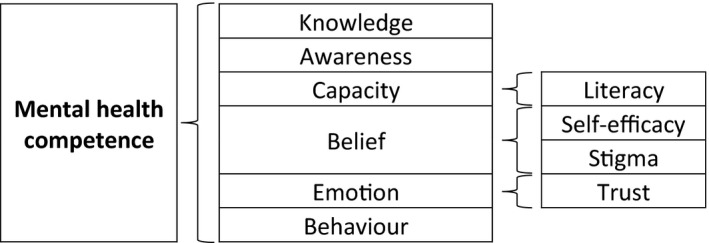
Intervention foci described by experts

Experts expressed that these training programmes can respond to nursing foci related to mental health competencies (Figure 2).

### 4.3 Outcomes assessment methods

The “Longitudinal evaluation with validated instruments based on knowledge in the nursing field” was shown to be important. Experts indicated that this longitudinal assessment should occur before the intervention, at the end and at the follow‐up “with a timing to be decided” (E4). In this measurement of outcomes, they suggested the use of validated assessment instruments. Among the instruments validated for the Portuguese population, the following were listed: the QuALiSMental (Loureiro, 2015), the MentaHLiS (Rosa et al., 2016) and the Mental Health Literacy Scale (Loureiro, 2018). Recognizing that these assessment instruments have a pathogenic perspective of mental health literacy, the development of valid instruments based on indicators of the Nursing Outcomes Classification (NOC) (Moorhead et al., 2018) was proposed.

### 4.4 Process assessment methods

The experts recommended a “Valid and adapted assessment of satisfaction for each training session.” The session assessment may involve observing participants’ behaviours (e.g. use of observation grids/scales, homework monitoring) and a verbal evaluation (e.g. use of Likert questions and scales, assignment of defining expressions). E3 indicated that “validated assessment instruments of training satisfaction could be searched.” More visual strategies should be considered. For example, E6 suggested, “emojis to classify the session.”

### 4.5 Participants

The intervention must have an “Implementation for classes with a nursing diagnosis, availability and willingness to learn.” Experts expressed the importance of intervening for classes (up to 30 students) without excluding participants from the target population. However, they considered necessary the existence of nursing diagnoses that justify the intervention and willingness and availability to learn in training programmes. For example, E3 stated, "There must be areas to work on, diagnoses related to (mental health) competencies." They also stressed that special attention must be paid to participants with physical, mental and social health problems.

### 4.6 Specific implementation context

These training programmes must have a “Classroom implementation during school time.” Several experts suggested the classroom as an implementation context. Due to the risk of infection (e.g. COVID‐19), E1 stressed the importance of performing the intervention in large rooms. E1 also hypothesized that “it may be advantageous to do (the intervention) outdoors,” but recognizes that “there will be many distractions.” The students’ usual academic period was recommended to carry out the intervention. E3 explained that “When it is included in school hours, we have more or less facilitated participation right from the start.”

### 4.7 Duration and frequency

According to the experts, these interventions should have “Regular sessions depending on school availability.” When carrying out the interventions in school time, E1 mentioned that the frequency of sessions should be adjusted to the length of the academic year. In turn, E1 and E7 highlighted the importance of negotiating the duration and frequency of the intervention, considering the availability of teachers and schools. Experts expressed that the duration of each session should be adjusted to the duration of the class (usually 45–90 minutes). E3 suggested, “we can create flexible plans from 45 to 90 minutes.” In addition, they proposed a break between sessions up to one week and the implementation of reinforcement/consultation sessions. E3 indicated that “(follow‐up) evaluation moments can be clustered in the reinforcement sessions.”

### 4.8 Intervention methods and strategies

This type of intervention should use a “Mixture of different training strategies and educational resources.” Experts recommended the use of expository, demonstrative, participatory strategies (such as quizzes, sharing and discussions), experimental learning methods (such as games, role‐plays and homework) and contact‐based education strategies (testimonials from reference persons). They highlighted the use of more active strategies, and E4 justified, “We must use many active methodologies especially because we are talking about first aid, of applying in practice knowledge that allows us to help others in a specific situation of suffering.” They also stated that it is important to use educational materials, audiovisual resources (such as videos, movies and music) and technologies (such as computers and mobile phones, the Internet and websites).

In addition, “Awareness sessions for the school community” should be considered. Although the training target is adolescents, experts suggested clarification and awareness sessions on the subject for parents, teachers and non‐teaching staff.

### 4.9 Contents

Experts described the “Components of mental health literacy” as topics to be addressed in training programmes. The contents suggested by the experts are summarized in Table 2.

**TABLE 2:** General contents of training programmes described by experts

Mental Health Literacy Components	Contents
Knowledge of effective self‐help strategies	Concept of health and mental health Coping strategies
Knowledge of how to prevent mental disorders	Risk and protective factors for mental health problems Model by Dahlgren and Whitehead (1991)
Recognition of when a disorder is developing	Mental problems and disorders Experience of mental problems and stigma
Knowledge and skills to give first aid and support to others	First aider role Actions to aid others
Knowledge of help‐seeking options and treatments available	Help resources available, access and referral for help Facilitating factors and barriers for seeking help

They emphasized the importance of addressing “Mental health nursing problems.” The problems described were cognitive, emotional, behavioural and relational (Table 3).

**TABLE 3:** Mental health problems to be addressed in training programmes, according to experts

Type of Problems	Problems Listed
Cognitive problems	Hallucination, Impaired thinking, Delirium, Suicidal ideation, Confusion, Negative self‐image, Disturbed personal identity, Impaired concentration, Impaired attention
Emotional problems	Anxiety, Nervousness, Sadness, Depressed mood, Grief, Loneliness, Fear, Stress, Fatigue, Exhaustion, Distress, Despair, Jealousy, Guilt, Frustration, Insecurity, Envy, Anger, Powerlessness, Suffering, Shame, Euphoria, Lack of hope, Lack of trust, Lack of pride, Lack of pleasure
Behavioural problems	Self‐care deficit, Impaired sleep, Impaired eating behaviour, Impaired eating behaviour [Anorexia], Bulimia, Compulsive eating behaviour, Compulsive behaviour, Impaired exercise behaviour, Substance abuse, [Video game] abuse, [Internet] abuse, Aggressive behaviour, Violence, Self‐destructive behaviour, Self‐mutilation, Attempted Suicide, Disorganized Behaviour
Relational problems	Impaired Communicating Act, Impaired Socialization

The “dynamic mental health first aid plan” was exposed as an important content to be considered. E4 explains that "It is a key principle that the action plan is dynamic and that the order of steps can be changed depending on who we have in front of us." The aid actions to be taught are listed in Table 4.

**TABLE 4:** Mental health first aid actions described by experts

General action plan	Specific actions
Approach the person and assess the situation	Introduce yourself; Explain the purpose of the aider's presence; Ensure confidentiality; Provide presence; Be present; Provide help; Actively listening with an expression of interest, respect, understanding and without judgement; Assess the person's cultural background; Observe behaviour; Coordinate behaviour with the person being helped; Physically approach the person, ensuring their own safety; Accompany the person to a quiet, safe, comfortable place that allows privacy; Ask about the situation; Ask about the problem, causes, aggravating and mitigating factors, and consequences
Assist and encourage the person to use self‐help strategies	Assist in identifying the mental health problem; Inform about the mental health problem; Assist in identifying adaptive strategies; Encourage the use of adaptive strategies; Praise adaptive strategies already in use; Encourage abandonment of maladaptive strategies
Assist and encourage the person to seek formal and informal help	Assist in the seeking for informal and formal help; Inform about the role of help resources; Accompany in the seeking for informal and formal help; Asking for help in an extreme situation; Encourage the seeking for informal and formal help
Take care of oneself first aider)	Use adaptive self‐help strategies

Therefore, internal and external resources of people with mental health problems can be mobilized in this process. Self‐help strategies that experts have indicated include healthy lifestyles (e.g. establishing routines, leisure activities). Help from family, social network (e.g. friends, colleagues), school (teaching and non‐teaching staff), local health services (doctors, psychologists, nurses, self‐help/mutual aid groups) and distance health services (telephone helplines, virtual therapies) were highlighted.

## 5 DISCUSSION

This study aimed to explore nurses’ perspectives on Mental Health First Aid Training Programmes for adolescents in Portuguese upper secondary schools. Our results expressed the characteristics of the facilitators and participants of these interventions. The experts listed intervention foci and evaluation methods that should be considered in training programmes. They identified the specific implementation context, duration and frequency of sessions. In addition, they gave their views on intervention methods and contents of training programmes.

Experts described the competencies of nurses who perform these interventions. They can be found in mental health nurses (International Council of Nurses [ICN], 2009; Regulation No, 2018. 515/2018). The Ordem dos Enfermeiros declares that a mental health nurse "has a high level of knowledge and self‐awareness" and "provides psychoeducational care (…), mobilising the context and dynamics of individuals, families, groups or communities" (Regulation No, 2018 515/2018, pp. 21427). Nurses with other specialities (e.g. community health, paediatrics) have some of the competencies described (International Council of Nurses [ICN], 2009). Therefore, they can be considered in the co‐dynamization of these interventions. The existence of co‐facilitators in training programmes was valued in this study. Professionals from other disciplines (e.g. psychiatrists, psychologists, teachers) and contexts can also be included as they are important professionals in the intervention of mental health problems (Kitchener et al., 2017).

The intervention foci expressed by the experts were related to mental health competence. According to Salman et al. (2020), competence can be improved with training and involves knowledge, skill, attitude, performance and task execution. Experts recommended an evaluation of interventions over time with valid and appropriate methods. Longitudinal evaluation of the results allows us to understand the effectiveness and usefulness of the intervention (Richards & Hallberg, 2015). Outcome assessment instruments with a non‐medicalized perspective of mental health literacy are needed to accompany these training programmes (Costa et al., 2020). Indicators of the Nursing Outcomes Classification (NOC) can be developed and used to assess the outcomes of an intervention promoted by nurses (Sampaio et al., 2017). Alternatively, another consensus among nurses can be considered. The assessment of satisfaction in each session allows identifying aspects to be improved in the intervention (Richards & Hallberg, 2015). The validity of the assessment instruments used is fundamental as it reflects the degree to which an instrument measures the construct it intends to measure (Mokkink et al., 2010).

This study highlighted the implementation of training programmes for classes with a nursing diagnosis, availability and willingness to learn. In schools, students are usually organized into classes. If a class is already well consolidated, it may already find itself in a phase of “work and productivity” (Åkerlund et al., 2021). Therefore, implementing training programmes in these pre‐existing groups can enhance learning. Moreover, the interventions performed by nurses require participants to have nursing diagnoses (International Council of Nurses [ICN], 2008). Nursing diagnoses are labels assigned to patients by nurses, in which outcomes are sought (International Council of Nurses [ICN], 2008). Motivation has also been described as a key factor for learning (Grossman & Salas, 2011).

Experts recognized that interventions should be implemented in the classroom during school time. The classroom is a controlled setting with more limited stimuli (Guajardo et al., 2019; Hart et al., 2016, 2020). Health interventions could be carried out at school times or during rest periods. School times are predetermined periods for learning and curricular activities (Normative Order No, 2018 10‐B/2018). Therefore, adherence to health training programmes can be promoted using regular teaching hours. Experts indicated that the implementation of interventions depends on the availability of schools. Flexible schedules can facilitate the implementation of interventions in different contexts (Mendenhall & Jackson, 2013). Moreover, breaks between sessions and/or supplementary meetings allow the participants to recognize and apply concepts in their daily lives and review their experiences in repeated contact with the facilitators.

According to the experts, different training strategies and educational resources can be used. Landøy et al. (2020) explain that the choice of teaching methods should be made, considering the training objectives, the skills of trainees and trainers and the information content to be mastered. These authors express that all teaching methods have advantages and disadvantages. Therefore, mixing methods can take advantage of each of them. Portuguese adolescents also expressed a preference for face‐to‐face interventions and improved learning through various training strategies (Costa et al., 2021).

Moreover, the experts suggested the involvement and awareness of the entire school community about mental health issues. Schools, families and community environments play key roles in adolescents’ learning (Spier et al., 2018). In the study by Costa et al. (2021), the adolescents agreed on the importance of raising awareness in the school community.

According to the experts, the training programmes should address the different components of mental health literacy described by Jorm (2012). They highlighted mental health nursing problems (cognitive, emotional, behavioural and relational). The listed problems can be found in nursing classifications, such as the International Classification for Nursing Practice (International Council of Nurses [ICN], 2019) and NANDA International (Herdman et al., 2021). Nurses can teach the community about their autonomous areas of intervention. Finally, the first aid behaviours proposed by the experts represent the action plan reported by Costa et al. (2020). As a result of a broad synthesis of evidence, this action plan can respond to different mental health problems. A dynamic action plan (e.g. with actions of flexible frequency and order) may be adequate considering that the people to be helped may find themselves in very different situations. For example, in the presence of a person with a mental health problem that manifests dysfunction, a first aider can assist in voluntarily seeking professional help. The same aider may need to ask for professional help for a person, without their consent, when identifying potential risk of harm to the person or third parties (Law No, 1998 36/1998). It should be noted that the perspective of experts about the contents of training programmes is in line with the opinion of Portuguese adolescents (Costa et al., 2021).

### 5.1 Limitations

The results are not generalizable, as the sampling was non‐probabilistic. Furthermore, the characteristics of the intervention facilitators were similar to those of the mental health nurses. However, the value of this finding can be considered limited, as the experts were mental health nurses.

## 6 IMPLICATIONS FOR PRACTICE

This study provides an exploratory view of the perspective of nurses about Mental Health First Aid Training Programmes for adolescents in Portuguese upper secondary schools. Mental health nurses were recognized as preferred facilitators of interventions. Thus, the potential autonomous role of the mental health nurse in the education of populations was emphasized. This evidence can sensitize and encourage mental health nurses to lead multidisciplinary teams in planning, implementing and evaluating these interventions. Nursing education curricula should enable mental health nurses to implement training programmes consistently. In addition, all “non‐mental health” nurses can be made aware of this intervention, as they can assist in carrying it out or even participate in helping people with mental health problems.

This report provides recommendations that can contribute to the development of this type of intervention. However, the development of a valid training programme requires broad consensus on its characteristics. For instance, statements regarding the opinions of adolescents and nurses (participants and facilitators of the intervention respectively) can be submitted to Delphi studies. The perspective of potential co‐facilitators of these training programmes can also be explored. Professionals from different specialities and disciplines should have the opportunity to express their role / contribution to the implementation of this type of training programmes led by mental health nurses. In addition, the lack of instruments valid to assess these healthier interventions was reported. The identification of assessment instruments requires literature reviews. If necessary, the construction of assessment tools and/or the development of studies regarding their psychometric properties and cultural adaptations should be considered.

The body of knowledge in nursing was highlighted, as training programmes can address mental health nursing problems. In this way, the participants learn to identify, assist and/or refer people with mental health problems in an adequate and timely manner, without any mistaken attribution of “medical labels.” This approach can be a direction for improving populations’ mental health literacy and combating the psychiatrization of human suffering.

## 7 CONCLUSIONS

Mental health nurses discussed multiple characteristics of Mental Health First Aid Training Programmes for adolescents in Portuguese upper secondary schools. They considered that these interventions could focus on the mental health competencies of students and classes. Valid and longitudinal evaluations of interventions allow representing their processes and outcomes. In regular sessions, different training strategies and educational resources can be used to teach adolescents to help people with mental health nursing problems. A more active role in mental health issues can be promoted at school.

## 8 RELEVANCE STATEMENT

Nurses play an important role in educating communities about mental health issues. They can promote Mental Health First Aid Training Programmes in upper secondary schools, enabling adolescents to help people with mental health problems. This paper gave voice to mental health nurses and shared their views on the topic. They expressed that a mix of educational methods should be used in the classroom to address the components of mental health literacy, various mental health nursing problems and a first aid plan. Longitudinal evaluation of interventions requires appropriate instruments. The experts’ recommendations can facilitate the development of autonomous interventions in this area.

## AUTHOR CONTRIBUTION

All authors made substantial contributions to the conception and design of the work. The first two author contributed to acquisition, analysis and interpretation of data and drafting the work. All authors critically reviewed the intellectual content, approved the final version to be published and agreed to account for all aspects of the work.

## ETHICS STATEMENT

This study is part of a project that received ethics approval from the Bioethics Committee of the University of Barcelona (Institutional Review Board: IRB00003099).

## Supporting information

Supplementary Material

Supplementary Material

## Data Availability

The data sets generated and/or analysed during the current study are not publicly available due to ethical considerations, but are available from the corresponding author on reasonable request.
