# Supplementary material for: Nurses’ perspective about the Mental Health First Aid Training Programmes for adolescents in upper secondary schools: A focus group study
Source: J Psychiatr Ment Health Nurs. 2022 Feb 8;29(5):721–31. doi: 10.1111/jpm.12823 (PMC9541295; doi:10.1111/jpm.12823)
Supplement: Supplementary file 1 — Supplementary Material [file JPM-29-721-s001.docx]

**Interview Guide** (translated from Portuguese to English)

**Interviewers presentation**: Good morning/afternoon! My name is *(interviewer's name/first author)*. I am a Nurse at the *(institution where the interviewer works)* and Doctoral Student at the *(institution where the interviewer studies).* I am accompanied by *(co-interviewer's name/*second author), Professor at the *(institution where the interviewer works)*.

**Study presentation:** As part of my PhD, we are conducting a study that aims to explore the nurses’ perspective about the Mental Health First Aid Training Programmes for adolescents in Portuguese upper secondary schools. These educational programmes empower adolescents to aid people with mental health problems.

**Presentation of the objective of the interview:** We will discuss the characteristics of these programmes, that is, their facilitators, intervention foci, outcomes and process assessment methods, participants and specific context of implementation, duration and frequency, intervention methods and strategies, and contents.

**Acknowledgement for presence and availability:** Thank you for your availability. Your help is essential for us.

**Explanation of how the meeting works:** In this meeting I will ask questions, giving you the opportunity to give your opinion. As explained in the informed consent, the meeting will be recorded to allow future transcription.

***(Confirm interview recording)***

**Involvement Questions:**

Do you want to introduce yourself briefly? First and last name and main affiliation.

How are you since the last meeting?

Since the last meeting, did you have any new ideas about these training programmes?

| **Exploratory Questions:**   1. What should be the characteristics of the nurses who promote the Mental Health First Aid Training Programmes for adolescents in Portuguese upper secondary schools?    1. How many facilitators are needed to implement a training programme?    2. Should facilitators other than nurses be considered? 2. What do you consider to be the most sensitive nursing foci of these intervention programmes?    1. How should the results obtained in these intervention foci be evaluated?    2. When should the results obtained in these intervention foci be evaluated? 3. What aspects should be considered when evaluating the intervention process?    1. How should the process evaluation be carried out?    2. When should the process assessment be carried out? 4. What requirements must participants have to participate in the programmes?    1. Are there any contraindications or precautions we should consider? If yes, which ones?    2. What is the appropriate number of participants per implementation?       1. If two or more, how should the participants be grouped? 5. What is the specific context of the school where the programmes should be implemented? 6. What duration (total and per session) and frequency should the interventions have?    1. What should be the interval between sessions (if applicable)? 7. What training strategies do you consider pertinent to use? 8. What contents should be covered in training programmes?    1. What mental health (nursing) problems should be addressed?    2. What steps should the “action plan” of mental health first aid have? |
| --- |

**Exit Question:** Do you have anything else to add to what was said during this meeting?

**Thanks and inform about the new contact:** I would like to thank you once again for your availability. I will contact you again, via e-mail, to return the transcript of this meeting to you. Is it possible? Please feel free to make comments or corrections.

**If a new meeting is needed:** In addition, I will send you a new form to check your availability for scheduling a new meeting.
